# Effects of constraint-induced movement therapy on activity and participation after a stroke: Systematic review and meta-analysis

**DOI:** 10.3389/fnhum.2022.987061

**Published:** 2022-12-05

**Authors:** Joyce Araújo de Azevedo, Felipe Douglas Silva Barbosa, Valquiria Martins Seixas, Kelly Regina Dias da Silva Scipioni, Priscila Yukari Sewo Sampaio, Daniel Marinho Cezar da Cruz, Daniele Piscitelli, Kevin K. Chui, Aristela de Freitas Zanona

**Affiliations:** ^1^Department of Occupational Therapy, Federal University of Sergipe, São Cristóvão, Sergipe, Brazil; ^2^Department of Occupational Therapy, Federal University of Bahia, Salvador, Bahia, Brazil; ^3^Department of Occupational Therapy, Federal University of Paraná, Curitiba, Paraná, Brazil; ^4^Departamento de Medicina, Universidade Federal de Sergipe, São Cristóvão, Sergipe, Brazil; ^5^School of Health, Leeds Beckett University, Leeds, United Kingdom; ^6^School of Physical and Occupational Therapy, McGill University, Montreal, QC, Canada; ^7^School of Medicine and Surgery, University of Milano-Bicocca, Milan, Italy; ^8^Department of Kinesiology, University of Connecticut, Storrs, CT, United States; ^9^Department of Physical Therapy, Waldron College of Health and Human Services, Radford University, Roanoke, VA, United States

**Keywords:** stroke, constraint-induced movement therapy, activities of daily living, social participation, occupational therapy

## Abstract

**Introduction:**

Hemiparesis is the main sensorimotor deficit after stroke. It can result in limitations in Activities of Daily Living (ADL) and social participation. Hemiparesis can be treated with behavioral techniques of intensive use of the affected arm, such as constraint-induced movement therapy (CIMT), however, it remains unclear whether motor improvement can lead to increases in the domains of activity and participation.

**Objective:**

Identify whether CIMT is superior to usual techniques to enhance activity and participation outcomes in stroke survivors.

**Methods:**

A systematic review with meta-analysis was conducted, based on the PRISMA guidelines. Search databases were: PubMed, LILACS, Embase, SciELO, Cochrane Library, Scopus, Medline, and Web of Science, with no language restriction. Meta-analysis was performed with Review Manager (version 5.3), significance level *p* ≤ 0.05.

**Results:**

A total of 21 articles were included for analysis. Superior effects were observed on motor function and performance in activities of daily living of individuals treated with CIMT. The outcomes measures utilized were: Fugl-Meyer Assessment (*p* = 0.00001); Wolf motor function test (*p* = 0.01); Modified Barthel Index (*p* = 0.00001); Motor Activity log (MAL) Amount of use (AOU) (*p* = 0.01); MAL Quality of movement (QOM) (*p* = 0.00001); Action Research Arm Test-ARAT (*p* = 0.00001); and FIM (*p* = 0.0007).

**Conclusion:**

Our results show that CIMT results in more significant gains in the functional use of the upper limb in ADL and functional independence, demonstrating superior activity and participation results in stroke survivors when compared to conventional therapies.

## Introduction

Stroke is a matter of public health and the second leading cause of death in the world, with nearly 5.5 million deaths per year and affecting approximately 13.7 million people (Kuriakose and Xiao, 2020).

Several bodily systems can be compromised after a stroke. Deficits in motor skills can include hemiparesis, especially of the upper limb, as the main sensorimotor deficit after a stroke, affecting functional performance (Eichinger et al., 2020).

Physiologically, the function of the arm is impaired due to the imbalance of the transcallosal inhibitory circuits between the areas of the primary motor cortex of the cerebral hemispheres, in which there is an increase in the excitability of the less affected hemisphere. This leads to exaggerated inhibition of the most affected hemisphere, called imbalance of the inter-hemispheric competition (Nowak et al., 2009).

Among the techniques to promote rebalancing of inter-hemispheric competition after stroke, practice guidelines and meta-analyses (Winstein et al., 2016; Teasell et al., 2020) recommended the Constraint-induced movement therapy (CIMT) with a high level of scientific evidence. In the last years, studies have pointed to evidence that CIMT can be useful in promoting improvements in upper limb motor function (Bang, 2016; Ju and Yoon, 2018). The CIMT consists of the constraint of the non-hemiparetic upper limb for several hours a day, combined with a repetition of intensive shaping and task practices with the paretic upper limb (Wu et al., 2013). Furthermore, the transfer package (TP), a set of behavioral techniques to facilitate the transfer of therapeutic gains from the treatment setting to daily life, is also an important strategy of CIMT (Taub et al., 2013).

Most studies with CIMT analyzed the effects of the technique on body function and structure with excellent results (Gauthier et al., 2008; Hayner et al., 2010; Wang et al., 2011; Brunner et al., 2012; Abo et al., 2014; Ju and Yoon, 2018; Abba et al., 2020).

A previous study investigated the effects of CIMT on involvement in day-to-day activities and social participation, with results indicating possible improvements (Peurala et al., 2012) and improved patient-reported outcomes of health status (PROsHS) after stroke; however, for this outcome, CIMT does not seem to be superior to conventional therapy based on the current literature (Abdullahi et al., 2021). Similarly, in a broader Cochrane review published in 2015 (Corbetta et al., 2015), the authors found that CIMT was associated with limited recovery in motor impairment and motor function. On the other hand, such benefits did not satisfactorily translate to disability improvements. Corbetta et al. (2015) focused their Cochrane review on disability as a primary outcome, while the secondary outcomes were actual and perceived upper limb motor function, motor impairment, dexterity, and quality of life. Conversely, less attention was given to activity and participation outcomes, which are considered relevant for a successful post-stroke recovery (Noreau et al., 2004; Woodman et al., 2014). Additionally, considering the increased rate of publication on stroke recovery over the years (McIntyre et al., 2014) and the tendency to overestimate clinical trial results in neurorehabilitation (Tosatto et al., 2022), an updated systematic review may potentially be helpful for health professional to guide decision-making processes. Thus, the present study aims to provide an updated search from recent years and investigate if CIMT is superior to conventional therapies for improving the activity and participation in stroke survivors.

The World Health Organization (WHO) International Classification of Functioning, Disability and Health (ICF), describes activity as the performance of a task or action by an individual; and participation as its involvement in real situations of daily life (Engkasan et al., 2019). Activity can be considered the tasks a person performs during therapy to improve motor and/or sensory function. In other words, grasping a cup and lifting it to another place can be considered an activity, according to ICF, whereas participation is when the subject is doing real-life situations such as self-care occupations (ADL). Moreover, individuals after a stroke may have limitations in performing ADL and decreased participation in work and social life (Birke et al., 2020). In addition, restrictions on personal care during meals and household tasks, abandonment of leisure activities and hobbies can be identified (Lindgren et al., 2018). Therefore, this study aims to verify the following question: “Does constraint-induced movement therapy (CIMT) improve the activity and participation of stroke survivors compared to conventional therapies?”

## Methods

**FIGURE 1:**
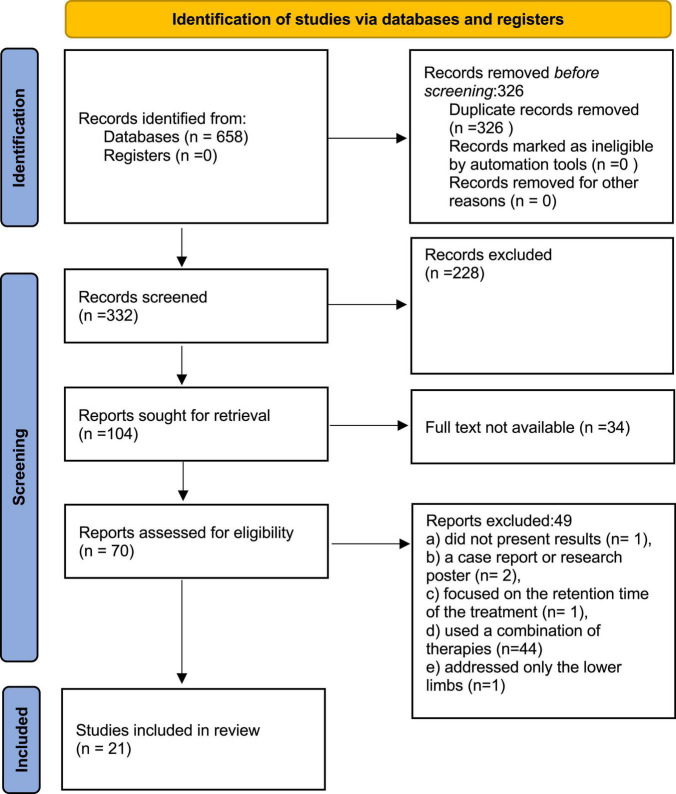
PRISMA flow diagram.

This research is a systematic review with meta-analysis, based on the Preferred Reporting Items for Systematic Reviews and Meta-Analyses (PRISMA) methodology (Galvão et al., 2015). Figure 1 describes the flowchart of the method applied.

### Eligibility criteria

As inclusion criteria, we considered randomized and non-randomized clinical trials, whose outcomes focused on activity/participation or functionality or occupational performance (the ability to plan and carry out roles, routines, tasks and sub-tasks for the purpose of self-maintenance, productivity, leisure and rest in response to demands of the internal and/or external environment). In their objectives, these studies must have also included a comparison between CIMT and any conventional therapy. A population of adult stroke survivors at any stage of the disease (acute, subacute or chronic) were included. We included articles available only with full text, published in journals, with no language restriction. As exclusion criteria letters to the editor, systematic (narrative) reviews, opinion pieces and experimental designs with animals were not considered for this review.

### Searching

At first, a guiding clinical research question was formulated using the PICO framework. Then, the search terms were defined based on the guiding question, followed by a check to determine whether the terms corresponded to the Medical Subject Headings (MeSH) classification. The main terms and entry terms were chosen for further search in the databases. The Boolean operators “AND” and “OR” were used. The following keywords were used: *stroke; constraint-induced movement therapy; conventional therapy; activities of daily living; participation* (Supplementary Appendix A).

In the first stage, the search for articles was carried out in the following databases: PubMed, LILACS, Embase, SciELO, Cochrane Library, Scopus, Medline and Web of Science during the months of December/2020 and January/2021. Selected publications between 2000 and 2020 were included in our review. Manual searches were also carried out in Google Scholar using the references of selected articles from databases, in theses and dissertations.

### Selection process

Two independent researchers searched and then jointly decided which studies would be included based on the eligibility criteria for this study. All articles were reviewed based on their titles and abstracts. Any discrepancies regarding record inclusion were resolved through consensus or consultation with a third reviewer author (AFZ and JAA). Duplicate publications were excluded. Then in the next step, the included articles were read in full for data extraction to compose the results matrix. Studies from the same research team were included because the CIMT protocols were different, and the study’s sample size was different. In addition, every effort was made to include articles in this review, paid articles or unavailable for download were requested via email to the librarian at the University where the study was conducted or direct email to the authors’ articles of interest.

### Data collection process

The data extracted from the articles to make the result matrix were: author’s name, publication year, study aims/objectives, number of participants, main results, dosage, and conclusion (Supplementary Table 1).

### Data analysis

For qualitative analysis, the parameters of the PEDro Scale were used. This scale is an instrument to assist users of the PEDro database to rapidly detect studies with internal validity and relevant information for interpreting the results. This tool consists of 11 criteria, classified as “no” or “yes” if the item is satisfied (Table 1). In addition, the score is calculated from criterion 2 until 11, as the first criterion refers to external validity (Shiwa et al., 2011).

**TABLE 1:** Qualitative analysis of studies on the PEDro scale.

	Study	1	2	3	4	5	6	7	8	9	10	11	Total
1	Lin et al., 2007	✓	✓	✓	✓	✓		✓	✓		✓	✓	9
2	Bang et al., 2018	✓	✓	✓	✓			✓	✓		✓	✓	8
3	Wu et al., 2013	✓	✓	✓				✓	✓		✓	✓	7
4	Brunner et al., 2012	✓	✓	✓	✓			✓	✓		✓		7
5	Lin et al., 2009a	✓	✓			✓		✓	✓		✓	✓	7
6	Lin et al., 2009b	✓		✓				✓	✓		✓	✓	6
7	Hayner et al., 2010	✓	✓					✓	✓		✓	✓	6
8	Smania et al., 2012	✓		✓				✓	✓	✓	✓		6
9	Lin et al., 2010	✓			✓				✓		✓	✓	5
10	Bang, 2016	✓		✓				✓			✓	✓	5
11	Abo et al., 2014	✓	✓					✓	✓		✓		5
12	Kim et al., 2018	✓			✓	✓			✓		✓		5
13	Yoon et al., 2014	✓	✓					✓	✓			✓	5
14	Page et al., 2009	✓	✓					✓			✓		4
15	Wang et al., 2011	✓	✓						✓		✓		4
16	Wu et al., 2007	✓							✓		✓	✓	4
17	Abba et al., 2020	✓	✓					✓	✓				4
18	Ju and Yoon, 2018	✓	✓						✓				3
19	Gauthier et al., 2008		✓						✓		✓		3
20	Kim and Chang, 2018	✓							✓		✓		3
21	Atler et al., 2015	✓							✓				2

1: Eligibility criteria and origin of participants; 2: Random allocation; 3: Secret allocation; 4: Similarity between groups; 5: Blind participants; 6: Blind therapists; 7: Blind evaluators; 8: Adequate follow-up; 9: Intent to treat; 10: Inter-group comparisons; 11: Point estimates and variability.

For quantitative analysis, the results were shown as mean and standard deviation analysis, with 95% confidence intervals (CI), presented using forest plots. For the meta-analysis, groups were allocated into control (conventional therapy, i.e., exercises and functional activities commonly applied in clinical practice) or experimental (CIMT with or without conventional therapy).

Upper limb motor function and activity, and participation were evaluated. Heterogeneity was quantified by the Cochran test (Ch2) which identified the inconsistency (percentage of the total variation of the studies due to heterogeneity) of the effects by the I2 statistics. A random analysis model was used; considering that the levels of a factor in a population were randomly captured, it was assumed that the individual effects were randomly distributed around an average. Funnel plots were used to assess publication bias. The Review Manager statistical program (version 5.3, Informer Technologies, Inc.) was used, with a significance level of *p* ≤ 0.05.

## Results

According to the search method, 658 articles were found. After eliminating duplicates, 332 were analyzed by reading the title and abstract. Of these, 70 articles were read in full. Of these, 49 articles were excluded for not meeting all the inclusion criteria. At the end of this process, 21 articles were included in the systematic review (see flowchart, Figure 1). Characteristics of the included studies were all in the English language, with a sample varying from 10 to 76 individuals, samples age ranging from 18 to 90 years old, and a time ranging from 1 to 36 months since the onset of stroke (ischemic or hemorrhagic). Supplementary Table 1 summarizes the characteristics of the included studies.

Regarding treatment techniques, the studies addressed CIMT; both in association with CIMT and trunk restriction (Bang, 2016, Bang et al., 2018); CIMT plus eye patching (Wu et al., 2007) mental practice (Page et al., 2009; Kim et al., 2018) and mirror therapy (Yoon et al., 2014), as well as techniques that were used in another group as a form of control: proprioceptive neuromuscular facilitation (Abba et al., 2020); bilateral treatment of equal intensity (Lin et al., 2009a; Hayner et al., 2010; Brunner et al., 2012); traditional therapies such as stretching, weight-bearing, balance, and functional task performance (Lin et al., 2007, 2009b; Wang et al., 2011; Smania et al., 2012; Kim and Chang, 2018); neurodevelopmental techniques (Gauthier et al., 2008); Low-Frequency rTMS and Occupational Therapy (Abo et al., 2014); and mirror therapy (Ju and Yoon, 2018).

With regard to the assessments/outcome measures examined, the majority of the studies used the following three assessments: 85.7% used the Mini Mental State Examination, 66.6% the Motor Activity Log and 52.3% the Modified Ashworth Scale. In addition to these, 47.6% of the studies used the Fugl Meyer assessment; 38% the Brunnstrom Scale; 33.3% the Wolf Motor Function Test (WMFT); 23.8% the Barthel Index; 19% used the Functional Independence Measure and 19% used the Action Research Arm Test (ARAT).

Stroke Impact Scale, Canadian Occupational Performance Measure, Kinematic analysis, Nine Hole Peg Test and Visual Analog Scale were used only in 9.5% of studies; and the Manual Function Test (MFT) was used in 14.2% of the articles. The other assessment instruments included in 4.7% of the studies were the Profile of Daily Experiences of Pleasure, Productivity and Restoration (PPR Profile); Motor Activity Diary; Maximum Elbow Extension Angle during Reach (MEEAR); European Stroke Scale; Briggs-Nebes; motricity index; Goniometer; Nottingham Extended ADL Scale; Balance assessment developed by the author himself; Edinburgh Handedness Inventory; Catherine Bergego scale; 3D Motion Analysis; Motor evoked potential amplitude (MEP); Random Chinese Word Cancellation Test; randomversion of the SymbolCancellation Test; Motor Activity Diary Usage Quality Scale; Box and Block test; Grip strength test; Double Simultaneous Stimulation Test and Line Bisection Test.

Approximately 30.5% of all assessments used by the authors were activity-oriented and 13.8% focused on participation. The studies in this review included patients in three distinct phases of stroke: acute, subacute and chronic. The superior beneficial effects of CIMT were evidenced by improvements in motor function and occupational performance of the paretic upper limb when compared to the conventional treatments. In addition, 10 articles reported an improvement in activity (47.6%) and only 1 stated an improvement in participation (4.7%).

### Meta-analysis

**FIGURE 2:**
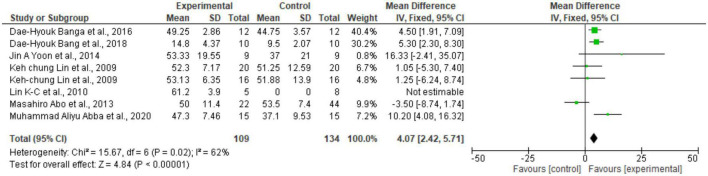
*Forest Plot* of the manual function assessed by the Fugl Meyer test. SD, standard deviation; CI, confidence interval.

**FIGURE 3:**
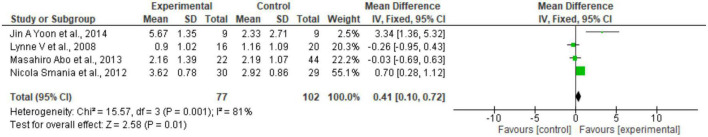
*Forest Plot* of the manual function assessed by the Wolf Motor Function test. SD, standard deviation; CI, confidence interval.

**FIGURE 4:**
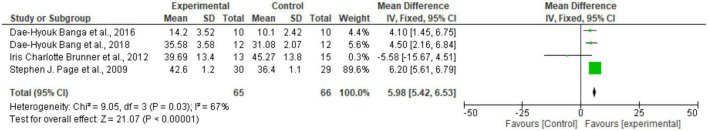
*Forest Plot* of the manual function assessed by the Action Research Arm Test. SD, standard deviation; CI, confidence interval.

**FIGURE 5:**
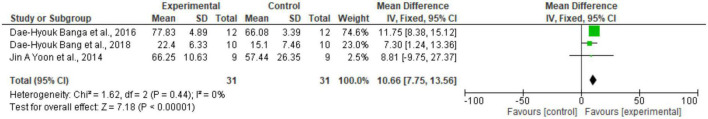
*Forest Plot* of the activity/participation assessed by the test Modified Barthel Index. SD, standard deviation; CI, confidence interval.

**FIGURE 6:**
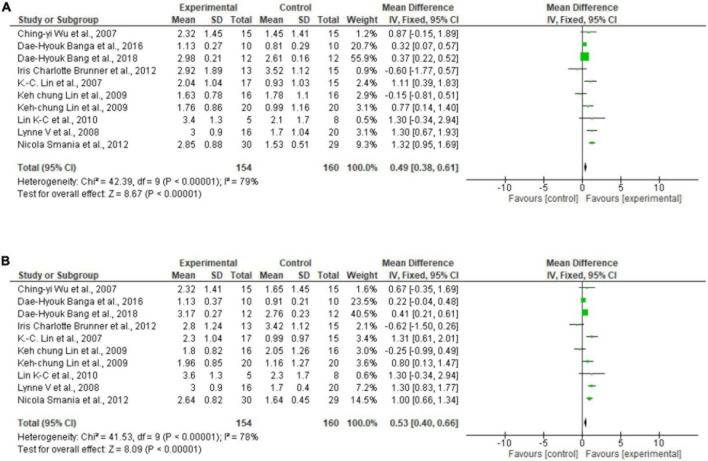
**(A)** *Forest Plot* of the activity/participation assessed by the test Motor Activity Log Amount of Use. **(B)** *Forest Plot* of the activity/participation assessed by the test Motor Activity Log Quality of Movement. SD, standard deviation; CI, confidence interval.

**FIGURE 7:**
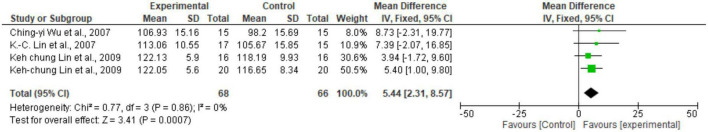
*Forest Plot* of the activity/participation assessed by the test Function Independence Measure. SD, standard deviation; CI, confidence interval.

Regarding the meta-analysis, 15 articles were included. Only the studies that presented, in their results, mean and standard deviation were added to the meta-analysis. The overall analysis found significant improvements in the experimental group (treatment) for the outcomes assessed by: Fugl-Meyer Assesment (mean difference = 4.07 Confidence interval—95%CI [2.42–5.71]; I^2^ = 62%, *p* = 0.00001) (Figure 2); Wolf motor function test (mean difference = 0.41 Confidence interval—95%CI [0.10–0.72]; I^2^ = 81%, *p* = 0.01) (Figure 3); Action Research Arm Test-ARAT (mean difference = 5.98 Confidence interval—95%CI [5.42–6.53]; I^2^ = 67%, *p* = 0.00001) (Figure 4) Modified Barthel Index (mean difference = 10.66 Confidence Interval—95%CI [7.75–13.56]; I^2^ = 0%, *p* = 0.00001) (Figure 5); MAL AOU (mean difference = 0.49 Confidence Interval—95%CI [0.38–0.61]; I^2^ = 79%, *p* = 0.01) (Figure 6A); MALQOM (mean difference = 0.53 Confidence interval—95%CI [0.40–0.66]; I^2^ = 78%, *p* = 0.00001) (Figure 6B); FIM (mean difference = 5.44 Confidence interval—95%CI [2.31–8.57]; I^2^ = 0%, *p* = 0.0007) (Figure 7). In addition, the funnel plots show symmetrical results and high concentration at the top of the pyramid, indicating a low risk of bias of studies included in the meta-analysis (Supplementary Appendix B).

## Discussion

The results demonstrate that the CIMT produced significant improvements with regard to the motor and function of the upper limb compared to some conventional therapies, such as mirror therapy, bilateral arm training, intensive conventional rehabilitation, mental practice, proprioceptive neuromuscular facilitation, conventional rehabilitation and conventional rehabilitation therapy (Lin et al., 2007, 2009b,^2010^; Wu et al., 2007; Page et al., 2009; Wang et al., 2011; Smania et al., 2012; Yoon et al., 2014; Atler et al., 2015; Ju and Yoon, 2018; Kim and Chang, 2018; Abba et al., 2020). In contrast, other results indicate that CIMT combined with another treatment technique (such as mental practice, auditory feedback, trunk restriction, and mirror therapy) results in a significant improvement compared to the therapy alone (Page et al., 2009; Yoon et al., 2014; Bang, 2016, Bang et al., 2018; Kim et al., 2018).

In the research in which CIMT was compared with Bilateral Arm Training (BAT), it was observed that both techniques are effective in terms of improving the general motor skills of the upper limb (Lin et al., 2009a). CIMT resulted in significant improvements in the functional use of the upper limb in daily activities and improved functional independence. However, in relation to the improvement of the proximal motor function, the BAT had a superior outcome (Lin et al., 2009a).

In addition to BAT, another technique that was superior when compared to CIMT was low-frequency repetitive transcranial magnetic stimulation (LF-rTMS) combined with intensive occupational therapy by improving movement and function (ADLs) of the entire upper limb (Abo et al., 2014). The researchers speculate that this superiority of the two techniques compared to CIMT can be related to the neuromodulation effects on direct functional reorganization in the brain, accelerating the plasticity process (Abo et al., 2014).

Few studies have related the results to the participation of stroke survivors in the context of daily living. Only one study used ICF participation as the primary research outcome (Atler et al., 2015). The research by Atler et al. (2015) investigated the relationships between participation, motor function and activity in 12 individuals with ischemic or hemorrhagic stroke with a mean time of 3 years since the stroke onset. Participants were asked to fill in the Functional Profile for three days a week to register the activities and evaluate their experiences of pleasure, productivity and restoration. For each activity recorded, the participant specified if the activity was performed alone or with another person and the location of the activity.

The motor function and activity results provide evidence that there is significant improvements after therapy in the upper limb. However, they also stated that this recovery is not retained after treatment, indicating that a single dose of CIMT is ineffective for long-term recovery in stroke survivors. Regarding participation, the findings of this study indicated that participants spent most of their time in IADL or household management activities and indicated that participants took longer to carry out household activities, consequently having less time for community activities and leisure. The authors concluded that improving motor skills does not reflect greater participation (Atler et al., 2015).

It is important to emphasize that it is essential to consider participation as an outcome measure to examine the impact after a stroke and collaborate with health professionals who also aim to improve the performance of daily activities of stroke survivors, especially in the engagement in ADL (Atler et al., 2015). The results of the study by Atler et al. (2015) reinforce that the improvement in motor function or activity performance does not necessarily result in greater participation in activities and life roles, suggesting the need for additional interventions that emphasize the reintegration of survivors into communities, rather than just focusing on restoring motor function.

Ju and Yoon (2018) conducted a study with 28 patients in the acute phase of stroke by comparing mirror therapy and CIMT to evaluate whether the improvement in limb function results in better ADL performance. Both groups participated in ADL training and self-exercise with therapeutic equipment. After the intervention, only the CIMT group had an improvement in the upper limb function, which influenced the performance of ADL, such as hygiene and feeding.

The results of another study evidenced a reduction in motor impairment and greater gains in functional capacity after CIMT, especially for self-care, locomotion and mobility (Lin et al., 2009b). The study reported a significant improvement in functional performance in individuals who received CIMT and suggested a better transference of treatment effects to daily life in patients allocated into this group (Wu et al., 2007).

Recently, another study verified the effects of CIMT on upper limb function and occupational performance of 14 stroke survivors, divided into experimental and control groups. The experimental group received CIMT and conventional rehabilitation therapy (CRT), which consisted of occupational and physical therapy; the control group received only CRT. The results showed that CIMT significantly improved occupational performance and upper extremity function compared to CRT (Kim and Chang, 2018).

Our meta-analysis indicated that CIMT protocols were superior and led to significant results for the motor function of the upper limb (Fugl-Meyer, WMFT and ARAT) and for the activity and participation outcomes (Modified Barthel, Motor Activity Log, and FIM). Thus, it is possible to conclude that the CIMT technique improves the ability of the upper limb and enhances the performance of ADL, resulting in an increased level of activity and participation. Methodologically, an aspect that should be noted when interpreting the present systematic review and meta-analysis findings pertains to the differences in primary endpoints of each included study. Indeed, the time frame when the outcome measures were collected may have a major influence on findings, e.g., between-group differences at 90 days and 6 months are more likely to be greater than differences at 1 year.

A different result found by Dromerick et al. (2009) states that the CIMT was as effective but not superior to an equal dose of traditional therapy during inpatient stroke rehabilitation equally. Furthermore, the authors stated that higher-intensity CIMT resulted in less motor improvement for up to 90 days, indicating an inverse dose-response relationship. However, it is possible that the VECTORs trial results were unfavorable because the authors did not consider the need for wrist and finger movements that are necessary for the patient to be eligible to receive CIMT. According to the authors, the inclusion criteria: “proximal UE voluntary activity indicated by a score of 3 on the upper arm item of the Motor Assessment Scale, but wrist and finger movement was not required.”

CIMT improves the use of the upper limb in activities and social participation because it induces plastic changes in the brain structure, consequently improving the upper limb’s performance in real life.

Following CIMT, the size of contrast-enhanced bilateral sensorimotor cortex on the voxel-based morphometry on T1-weighted magnetic resonance imaging scans increased. In addition, a correlation between the size of bilateral contrast-enhanced sensorimotor cortex and the degree of the functional recovery of the most affected upper limb has been reported (Gauthier et al., 2008). A sustained increased use of a body part leads to an increase in the brain’s cortical representation of that body part, while a decreased input reduces the representational zone of body part (Jenkins et al., 1990).

Three different explanations provided converging evidence that the group receiving CIMT therapy showed changes in gray matter in sensorimotor areas of the central nervous system and hippocampus, associated with improvements in spontaneous real-world arm function. In addition to the forced use of the upper limb during the therapy, the TP was found useful for improving learning and generalized movements to real-life environment. On the other hand, individulas whose therapy did not incorporate the TP, recovery was not the same as those of patients who used the forced upper limb in home settings (Gauthier et al., 2008).

The CIMT therapy produces functional changes in the brains of individuals with stroke in areas involving increases in the differential excitability, metabolic activity, and oxygen consumption of sensorimotor regions of the brain but also induces morphometric changes (Gauthier et al., 2008). Notably, increases were reported in the gray matter of the hippocampus. The hippocampus is known to be involved in learning and memory, which may explain the increase in quality and amount of use of the upper limb in ADL (Yamashima et al., 2004).

It is important to acknowledge that a Cochrane review was published in 2015 (Corbetta et al., 2015) to evaluate the effect of CIMT on upper limb recovery in individuals with stroke. The authors included more studies (n = 42) compared to the present work (n = 21). This difference may be explained by the chosen time frame, the database of the electronic searches and the eligibility criteria adopted. The Cochrane review searched fifty years back from 2015 and focused their work on upper limb disability, while we searched from 2000 to 2020, focusing primarily on activity and participation. We decided to update the topic to keep the evidence for researchers and clinicians up-to-date, considering that published works in the last years (i.e., from 2015) may lead to changes in the conclusions (Shojania et al., 2007). Our study showed that CIMT might improve activity and participation in stroke survivors. Clinicians should consider implementing CIMT when targeting activity and participation outcomes. Further research is warranted to investigate the effects of CIMT on ICF core sets, i.e., simultaneously changes in more than one ICF domain, and to determine the extent to which clinical and demographic characteristics of stroke survivors may influence the CIMT intervention.

## Conclusion

The studies included in this meta-analysis provide evidence that supports the effectiveness of CIMT in stroke survivors to improve motor function and functionality of the upper limb compared to conventional therapies. The findings from clinical investigations demonstrate that CIMT results in improvements in ADL performance and associated upper limb function, a reduction of motor impairment, and better transference of treatment effects to daily life, thus increasing the activity and participation of this population.

## Data availability statement

The original contributions presented in this study are included in the article/Supplementary material, further inquiries can be directed to the corresponding author.

## Author contributions

JA and AF: idea conception and text writing. JA, FB, and VS: search for studies and data extraction. AF and KS: data analysis. PS, DC, DP, and KC: text review. All authors contributed to the article and approved the submitted version.

## References

[B1] Abba M. A. Muhammad A. S. Badaru U. M. Abdullahi A. (2020). Comparative effect of constraint-induced movement therapy and proprioceptive neuromuscular facilitation on upper limb function of chronic stroke survivors. *Physiother. Q.* 28 1–5. 10.5114/PQ.2020.89809

[B2] Abdullahi A. Van Criekinge T. Umar N. A. Zakari U. U. Truijen S. Saeys W. (2021). Effect of constraint-induced movement therapy on persons-reported outcomes of health status after stroke: A systematic review and meta-analysis. *Int. J. Rehabil. Res.* 44 15–23. 10.1097/MRR.0000000000000446 33234842

[B3] Abo M. Kakuda W. Momosaki R. Harashima H. Kojima M. Watanabe S. (2014). Randomized, multicenter, comparative study of NEURO versus CIMT in poststroke patients with upper limb hemiparesis: The NEURO-VERIFY Study. *Int. J. Stroke* 9 607–612. 10.1111/IJS.12100 24015934

[B4] Atler K. Malcolm M. Greife C. (2015). A follow-up study on the relationship among participation, activity and motor function in survivors of stroke following constraint-induced therapy. *Disabil. Rehabil.* 37 121–128. 10.3109/09638288.2014.910560 24754600

[B5] Bang D. H. (2016). Effect of Modified Constraint-Induced Movement Therapy Combined with Auditory Feedback for Trunk Control on Upper Extremity in Subacute Stroke Patients with Moderate Impairment: Randomized Controlled Pilot Trial. *J. Stroke Cerebrovasc. Dis.* 25 1606–1612. 10.1016/J.JSTROKECEREBROVASDIS.2016.03.030 27062417

[B6] Bang D. H. Shin W. S. Choi H. S. (2018). Effects of modified constraint-induced movement therapy with trunk restraint in early stroke patients: A single-blinded, randomized, controlled, pilot trial. *NeuroRehabilitation* 42 29–35. 10.3233/NRE-172176 29400671

[B7] Birke G. Wolf S. Ingwersen T. Bartling C. Bender G. Meyer A. (2020). Protocol for a multicenter observational prospective study of functional recovery from stroke beyond inpatient rehabilitation - The Interdisciplinary Platform for Rehabilitation Research and Innovative Care of Stroke Patients (IMPROVE). *Neurol. Res. Pract.* 2:10. 10.1186/S42466-020-00056-2 33324916 PMC7650143

[B8] Brunner I. C. Skouen J. S. Strand L. I. (2012). Is modified constraint-induced movement therapy more effective than bimanual training in improving arm motor function in the subacute phase post stroke? A randomized controlled trial. *Clin. Rehabil.* 26 1078–1086. 10.1177/0269215512443138 22561098

[B9] Corbetta D. Sirtori V. Castellini G. Moja L. Gatti R. (2015). Constraint-induced movement therapy for upper extremities in people with stroke. *Cochrane Database Syst. Rev*. CD004433. 10.1002/14651858.CD004433.pub3 26446577 PMC6465192

[B10] Dromerick A. W. Lang C. E. Birkenmeier R. L. Wagner J. M. Miller J. P. Videen T. O. (2009). Very Early Constraint Induced Movement during Stroke Rehabilitation (VECTORS):A Single Center RCT. *Neurology* 73 195–201. 10.1212/WNL.0b013e3181ab2b27 19458319 PMC2715572

[B11] Eichinger F. L. F. Soares A. V. Noveletto F. Sagawa Júnior Y. Bertemes Filho P. Domenech S. C. (2020). Serious game for locomotor rehabilitation of hemiparetic stroke patients. *Fisioter. Em Mov.* 33:e003316. 10.1590/1980-5918.033.AO16

[B12] Engkasan J. P. Ahmad-Fauzi A. Sabirin S. Chai C. C. Abdul-Malek I. Z. Liguori S. (2019). Mapping the primary outcomes reported in Cochrane systematic reviews regarding stroke with the International Classification of Functioning, Disability and Health domains: Current trend and future recommendations. *Eur. J. Phys. Rehabil. Med.* 55 378–383. 10.23736/S1973-9087.19.05792-7 30961345

[B13] Galvão T. F. Pansani T. de S. A. Harrad D. (2015). Principais itens para relatar Revisões sistemáticas e Meta-análises: A recomendação PRISMA. *Epidemiol. Serv. Saúde* 24 335–342. 10.5123/S1679-49742015000200017

[B14] Gauthier L. V. Taub E. Perkins C. Ortmann M. Mark V. W. Uswatte G. (2008). Remodeling the Brain Plastic Structural Brain Changes Produced by Different Motor Therapies After Stroke. *Stroke* 39:1520. 10.1161/STROKEAHA.107.502229 18323492 PMC2574634

[B15] Hayner K. Gibson G. Giles G. M. (2010). Comparison of constraint-induced movement therapy and bilateral treatment of equal intensity in people with chronic upper-extremity dysfunction after cerebrovascular accident. *Am. J. Occup. Ther.* 64 528–539. 10.5014/AJOT.2010.08027 20825123

[B16] Jenkins W. M. Merzenich M. M. Och S. M. T. Allard T. Guic-Robles E. (1990). Functional reorganization of primary somatosensory cortex in adult owl monkeys after behaviorally controlled tactile stimulation. *J. Neurophysiol.* 63 82–104. 10.1152/jn.1990.63.1.82 2299388

[B17] Ju Y. Yoon I. J. (2018). The effects of modified constraint-induced movement therapy and mirrortherapy on upper extremity function and its influence on activities of dailyliving. *J. Phys. Ther. Sci.* 30:77. 10.1589/JPTS.30.77 29410571 PMC5788780

[B18] Kim H. Yoo E. Y. Jung M. Y. Kim J. Park J. H. Kang D. H. (2018). The effects of mental practice combined with modified constraint-induced therapy on corticospinal excitability, movement quality, function, and activities of daily living in persons with stroke. *Disabil. Rehabil.* 40 2449–2457. 10.1080/09638288.2017.1337817 28597693

[B19] Kim J.-H. Chang M.-Y. (2018). Effects of modified constraint-induced movement therapy on upper extremity function and occupational performance of stroke patients. *J. Phys. Ther. Sci.* 30 1092–1094. 10.1589/JPTS.30.1092 30154606 PMC6110232

[B20] Kuriakose D. Xiao Z. (2020). Pathophysiology and Treatment of Stroke: Present Status and Future Perspectives. *Int. J. Mol. Sci.* 21:7609. 10.3390/IJMS21207609 33076218 PMC7589849

[B21] Lin K. C. Chang Y. F. Wu C. Y. Chen Y. A. (2009a). Effects of constraint-induced therapy versus bilateral arm training on motor performance, daily functions, and quality of life in stroke survivors. *Neurorehabil. Neural Repair* 23 441–448. 10.1177/1545968308328719 19118130

[B22] Lin K. C. Wu C. Y. Liu J. Sen Chen Y. T. Hsu C. J. (2009b). Constraint-induced therapy versus dose-matched control intervention to improve motor ability, basic/extended daily functions, and quality of life in stroke. *Neurorehabil. Neural Repair* 23 160–165. 10.1177/1545968308320642 18981188

[B23] Lin K. C. Chung H. Y. Wu C. Y. Liu H. L. Hsieh Y. W. Chen I. H. (2010). Constraint-induced therapy versus control intervention in patients with stroke: A functional magnetic resonance imaging study. *Am. J. Phys. Med. Rehabil.* 89 177–185. 10.1097/PHM.0B013E3181CF1C78 20173425

[B24] Lin K. C. Wu C. Y. Wei T. H. Lee C. Y. Liu J. S. (2007). Effects of modified constraint-induced movement therapy on reach-to-grasp movements and functional performance after chronic stroke: A randomized controlled study. *Clin. Rehabil.* 21 1075–1086. 10.1177/0269215507079843 18042603

[B25] Lindgren I. Gard G. Brogårdh C. (2018). Shoulder pain after stroke - experiences, consequences in daily life and effects of interventions: A qualitative study. *Disabil. Rehabil.* 40 1176–1182. 10.1080/09638288.2017.1290699 28637154

[B26] McIntyre A. Richardson M. Janzen S. Hussein N. Teasell R. (2014). The evolution of stroke rehabilitation randomized controlled trials. *Int. J. Stroke* 9 789–792. 10.1111/ijs.12272 24621406

[B27] Noreau L. Desrosiers J. Robichaud L. Fougeyrollas P. Rochette A. Viscogliosi C. (2004). Measuring social participation: Reliability of the LIFE-H in older adults with disabilities. *Disabil. Rehabil.* 26 346–352. 15204486 10.1080/09638280410001658649

[B28] Nowak D. A. Grefkes C. Ameli M. Fink G. R. (2009). Interhemispheric competition after stroke: Brain stimulation to enhance recovery of function of the affected hand. *Neurorehabil. Neural Repair* 23 641–656. 19531606 10.1177/1545968309336661

[B29] Page S. J. Levine P. Khoury J. C. (2009). Modified constraint-induced therapy combined with mental practice: Thinking through better motor outcomes. *Stroke* 40 551–554. 10.1161/STROKEAHA.108.528760 19109542

[B30] Peurala S. H. Kantanen M. P. Sjögren T. Paltamaa J. Karhula M. Heinonen A. (2012). Effectiveness of constraint-induced movement therapy on activity and participation after stroke: A systematic review and meta-analysis of randomized controlled trials. *Clin. Rehabil.* 26 209–223. 10.1177/0269215511420306 22070990

[B31] Shiwa S. R. Costa L. O. P. Moser A. D. de L. Aguiar I. de C. (2011). PEDro: A base de dados de evidências em fisioterapia. *Fisioter. em Mov.* 24 523–533. 10.1590/S0103-51502011000300017

[B32] Shojania K. G. Sampson M. Ansari M. T. Ji J. Doucette S. Moher D. (2007). How quickly do systematic reviews go out of date? A survival analysis. *Ann. Intern. Med.* 147 224–233. 10.7326/0003-4819-147-4-200708210-00179 17638714

[B33] Smania N. Gandolfi M. Paolucci S. Iosa M. Ianes P. Recchia S. (2012). Reduced-intensity modified constraint-induced movement therapy versus conventional therapy for upper extremity rehabilitation after stroke: A multicenter trial. *Neurorehabil. Neural Repair* 26 1035–1045. 10.1177/1545968312446003 22661278

[B34] Taub E. Uswatte G. Mark V. W. Morris D. M. Barman J. Bowman M. H. (2013). Method for enhancing real-world use of a more affected arm in chronic stroke: Transfer package of constraint-induced movement therapy. *Stroke* 44 1383–1388. 10.1161/STROKEAHA.111.000559 23520237 PMC3703737

[B35] Teasell R. Salbach N. M. Foley N. Mountain A. Cameron J. I. Jong A. (2020). Canadian Stroke Best Practice Recommendations: Rehabilitation, Recovery, and Community Participation following Stroke. Part One: Rehabilitation and Recovery Following Stroke; 6th Edition Update 2019. *Int. J. Stroke* 15 763–788. 10.1177/1747493019897843 31983296

[B36] Tosatto D. Bonacina D. Signori A. Pellicciari L. Cecchi F. Cornaggia C. M. (2022). Spin of information and inconsistency between abstract and full text in RCTs investigating upper limb rehabilitation after stroke: An overview study. *Restor. Neurol. Neurosci.* 40 195–207. 10.3233/RNN-211247 35723125

[B37] Wang Q. Zhao J. L. Zhu Q. X. Li J. Meng P. P. (2011). Comparison of conventional therapy, intensive therapy and modified constraint-induced movement therapy to improve upper extremity function after stroke. *J. Rehabil. Med.* 43 619–625. 10.2340/16501977-0819 21603848

[B38] Winstein C. J. Stein J. Arena R. Bates B. Cherney L. R. Cramer S. C. (2016). Guidelines for Adult Stroke Rehabilitation and Recovery: A Guideline for Healthcare Professionals From the American Heart Association/American Stroke Association. *Stroke* 47 e98–e169. 10.1161/STR.0000000000000098 27145936

[B39] Woodman P. Riazi A. Pereira C. Jones F. (2014). Social participation post stroke: A meta-ethnographic review of the experiences and views of community-dwelling stroke survivors. *Disabil. Rehabil.* 36 2031–2043. 10.3109/09638288.2014.887796 24597937

[B40] Wu C. Y. Lin K. C. Chen H. C. Chen I. H. Hong W. H. (2007). Effects of modified constraint-induced movement therapy on movement kinematics and daily function in patients with stroke: A kinematic study of motor control mechanisms. *Neurorehabil. Neural Repair* 21 460–466. 17601803 10.1177/1545968307303411

[B41] Wu C. Y. Wang T. N. Chen Y. T. Lin K. C. Chen Y. A. Li H. T. (2013). Effects of constraint-induced therapy combined with eye patching on functional outcomes and movement kinematics in poststroke neglect. *Am. J. Occup. Ther.* 67 236–245. 10.5014/AJOT.2013.006486 23433279

[B42] Yamashima T. Tonchev A. B. Vachkov I. H. Popivanova B. K. Seki T. Sawamoto K. (2004). Vascular adventitia generates neuronal progenitors in the monkey hippocampus after ischemia. *Hippocampus* 14 861–875. 15382256 10.1002/hipo.20001

[B43] Yoon J. A. Koo B. Il Shin M. J. Shin Y. B. Ko H. Y. (2014). Effect of constraint-induced movement therapy and mirror therapy for patients with subacute stroke. *Ann. Rehabil. Med.* 38 458–466. 10.5535/ARM.2014.38.4.458 25229024 PMC4163585

