# Supplementary material for: Effects of constraint-induced movement therapy on activity and participation after a stroke: Systematic review and meta-analysis
Source: Front Hum Neurosci. 2022 Dec 5;16:987061. doi: 10.3389/fnhum.2022.987061 (PMC9760712; doi:10.3389/fnhum.2022.987061)

**APPENDIX A -** Search for each database

| Search strategy | Datebase | Number of articles |
| --- | --- | --- |
| *Stroke AND Constraint-induced Movement Therapy AND Conventional therapy AND Activities of daily living AND Participation* | *PubMed*  *LILACS*  *Embase*  *SciELO*  *Cochrane Library*  *Scopus*  *Medline*  *Web of Science*  **TOTAL** | 0  0  0  0  7  1  0  3  **11** |
| *Stroke AND Constraint-induced Movement Therapy AND Activities of daily living* | *PubMed*  *LILACS*  *Embase*  *SciELO*  *Cochrane Library*  *Scopus*  *Medline*  *Web of Science*  **TOTAL** | 16  1  63  1  72  124  0  77  **354** |
| *Stroke AND Constraint-induced Movement Therapy AND Participation* | *PubMed*  *LILACS*  *Embase*  *SciELO*  *Cochrane Library*  *Scopus*  *Medline*  *Web of Science* | 29  0  31  0  147  37  0  49 |
|  | **TOTAL** | **293** |

APPENDIX B

***Funnel Plot*  of the manual function assessed by the Fugl Meyer test**


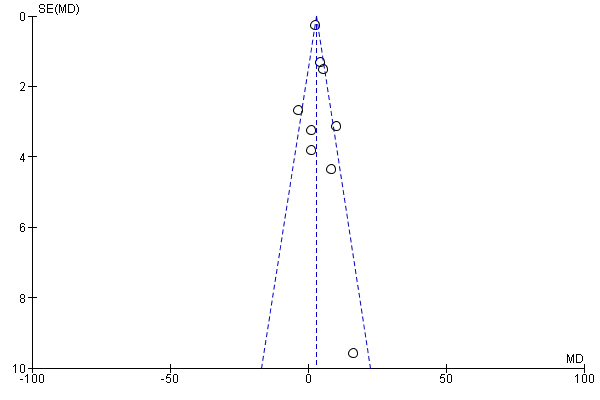


***Funnel Plot*** **of the manual function assessed by the Wolf Motor Function test**

**
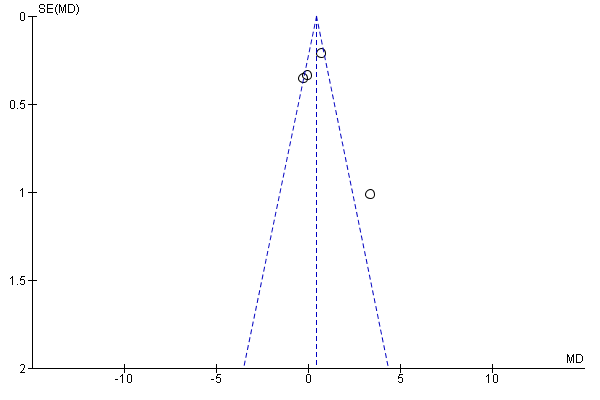
**

***Funnel Plot* of the manual function assessed by the Action Research Arm Test**


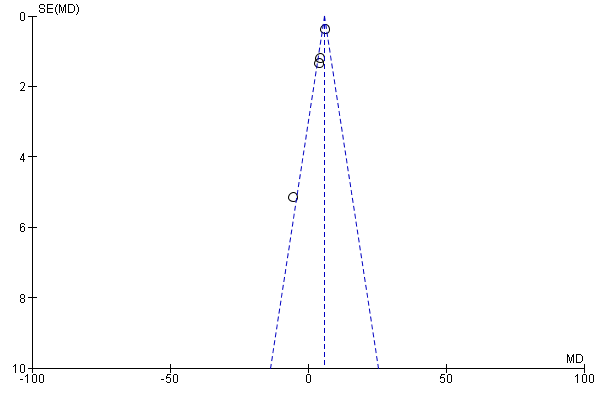


***Funnel Plot* of the activity/participation assessed by the test Modified Barthel Index**

**
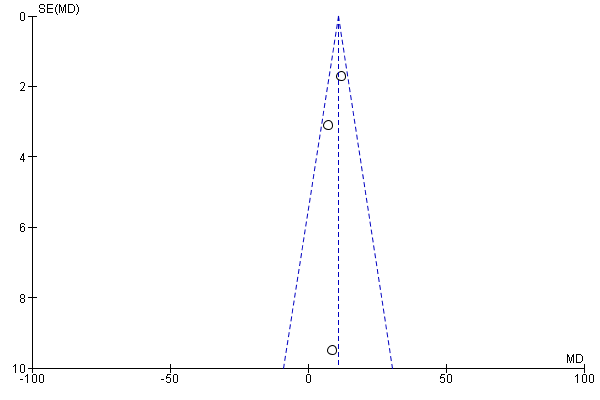
**

***Funnel Plot* of the activity/participation assessed by the test Motor Activity Log Amount of Use**

**
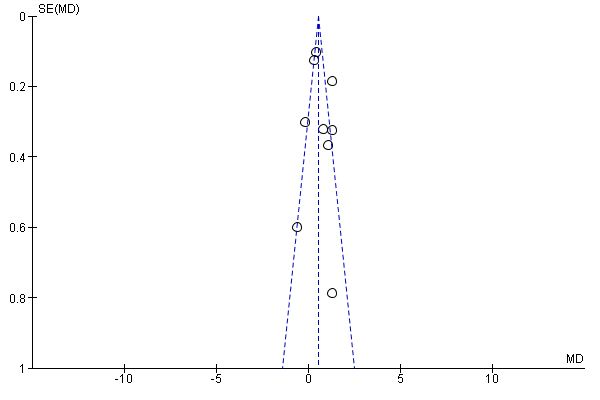
**

**Funnel Plot of the activity/participation assessed by the test Motor Activity Log Quality of Movement**


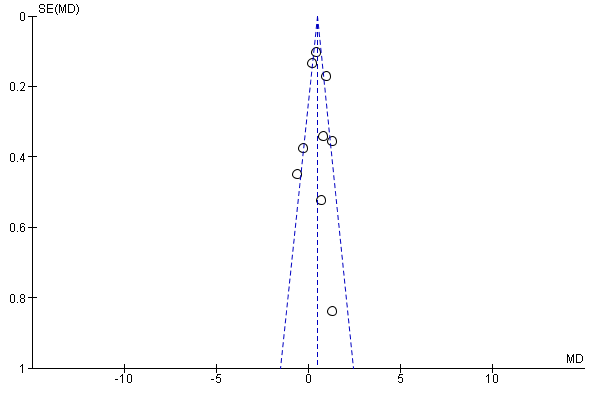


***Funnel Plot* of the activity/participation assessed by the test Function Independence Measure**


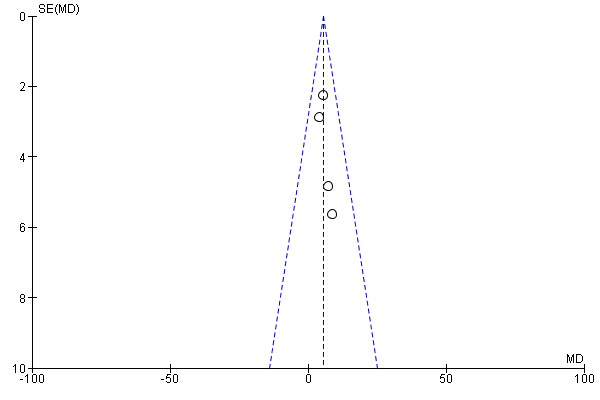

Supplement: Supplementary file 1 [file Data_Sheet_1.docx]
