# Supplementary material for: Effects of constraint-induced movement therapy on activity and participation after a stroke: Systematic review and meta-analysis
Source: Front Hum Neurosci. 2022 Dec 5;16:987061. doi: 10.3389/fnhum.2022.987061 (PMC9760712; doi:10.3389/fnhum.2022.987061)
Supplement: Supplementary file 2 [file Table_1.docx]

**Supplementary Table 1 - Outcomes on Body Function and Activity/Participation**

| **Authors/Year** | **Objective** | **Total Patients** | **Dosage** | **Results /Conclusion** |
| --- | --- | --- | --- | --- |
| Ching-Yi Wu; Tien-Ni Wang; Yu-Ting Chen; Keh-Chung Lin; Yi-An Chen; Hsiang-Ting Li; Pei-Luen Tsai, 2013. | To investigate the effects of combined CIMT-EP versus CIMT alone versus conventional training on improving functional performance. | 24 total, 7 in the CIMT + EP group, 8 in the CIMT group and 9 in the control group. | All Patients received 2 hours of therapy, 5 days a week for 3 weeks | - CIMT + EP and CIMT alone improved daily function more than the control intervention. CIMT was more beneficial in increasing left fixation points than the CIMT + EP intervention and in shortening reaction time than the control intervention.  - CIMT + EP and CIMT demonstrated similar beneficial effects on functional performance in patients' daily lives and differential effects on eye movement and range kinematics. CIT can improve eye movement and limb initiation, while CIMT + EP can facilitate pre-planned control and trunk control. |
| Keh-Chung Lin; Hsin-Ying Chung; Ching-Yi Wu; Ho-Ling Liu; Yu-Wei Hsieh; I-Hsuan Chen; Chia-Ling Chen; Li-Ling Chuan; Jung-Sen Liu; Yau-Yau Wai, 2010. | To investigate the effects of dCIMT in improving motor and daily functions compared to the CI group, and its neuroplastic effects on motor areas that are specific and different from the reorganization patterns after CI. | 13 total, 5 in the dCIMT group and 8 in the CI group. | All Patients received 2 hours per day, 5 days per  week, for three consecutive weeks. | - Compared with the CI group, the dCIMT group exhibited significantly greater improvements in motor and functional use of the affected arm. The dCIMT groups exhibited increased activation in the bilateral hemispheres immediately after the intervention, especially in the contralesional hemisphere during movement of the affected and unaffected hand.  - Preliminary results suggest that the functional improvements produced by dCIMT were accompanied by plastic reorganization of the brain, especially in the contralesional hemisphere, possibly via an ipsilateral motor pathway. |
| Yumi Ju; in-Jin Yoon, 2018. | To investigate whether changes in UE function directly impact improvement in ADL performance, comparing mirror therapy and mCIMT. groups. | 28 total | Mirror therapy and mCIMT including ADL training and self-exercise for both groups were provided 5 times a week,  60 min a session, and for 3 weeks | - Both therapy groups showed improvement in UE function. Only the mCIMT group showed increased UE function that was significantly correlated with ADL performance in the areas of hygiene, feeding, and dressings that primarily require hand and arm movements.  - Results suggest that attempting to voluntarily move the affected arm causes an increase in motivation and ultimately leads to functional improvement in ADL performance. |
| Stephen J. Page; Peter Levine; Jane C. Khoury, 2009. | Compare differences in motor outcomes associated with participation in the 2 interventions (mCIMT and MP). | 10 total, 5 in the mCIMT group and 5 in the mCIMT + MP group. | (1) structured therapy emphasizing affected arm use in functional activities 3 days/week for 10 weeks; and (2) less affected arm restraint 5 days/week for 5 hours. Both of these components were administered during a 10-week period. Subjects randomly assigned to the mCIT+mental practice experimental condition also received 30-minute mental practice sessions provided directly after therapy sessions | -All subjects exhibited considerable changes in outcome measures. These motor changes were clinically significant, conveying ability to perform skills not performed since before the subjects' strokes (eg, writing, buttoning a shirt).  - Modified restriction-induced therapy again produced clinically significant changes. However, the data also suggest that the addition of MP to the mCIMT protocol results in comparatively greater motor changes than mCIMT participation alone. |
| Keh-chung Lin; Ya-fen Chang; Ching-yi Wu; Yi-an Chen, 2009. | To compare the relative effects of dCIMT versus BAT versus control intervention on motor capacity, functional performance and quality of life. | 60 total, 20 in the CIMT group, 20 in the BAT group and 20 in the control group. | The distributed CIT group focused on restriction of movement of the unaffected hand by placement of the hand in a  mitt for 6 hours/day and intensive training of the affected UL  in functional tasks for 2 hours/weekday.  The BAT group concentrated on the simultaneous movements of both the affected and unaffected UL in functional  tasks in symmetric or alternating patterns for 2 hours/weekday  for 3 weeks  The control intervention group was designed to control for  the duration and intensity of patient-therapist interactions and  therapeutic activities (2 hours/day, 5 days/week, for 3 weeks) | - Both distributed CIMT and BAT improved overall UL and distal UL performance to a greater extent than the control intervention.  - Although both protocols improved UL's general motor skills, BAT was superior to distributed CIMT in improving proximal UL motor function. However, the beneficial effects of BAT may not translate into improvements in daily activity functions and quality of life. In contrast, distributed CIMT demonstrated greater gains in functional use of the affected UL in daily life and improved functional independence and quality of life over the BAT and control intervention |
| Keh-chung Lin; Ching-yi Wu; Jung-sen Liu; Yueh-tsen Chen; Chen-jung Hsu, 2009. | To examine the benefits of CIMT on different aspects of health in stroke survivors. | 32 total, 16 in the CIMT group and 16 in the control group. | Subjects in both groups received individualized, 2-hour  therapy sessions, 5 times per week for 3 weeks. | - The CIMT program reduced motor impairment to a greater extent and induced greater gains in functional capacity, especially for self-care, locomotion and mobility, than the control intervention. Patients in the CIMT group improved more in the physical aspects (ADL, mobility and hand function) of QOL than those in the control group.  - Experimental results suggest that the robust effects of intensive training of an affected limb on several important daily functions for home and community life are significant. |
| Qiang Wang; Jing-li Zhao; Qi-xiu Zhu; Jiang Li; Ping-ping Meng, 2011. | To compare the effectiveness of mCIMT with that of intensive conventional rehabilitation in motor recovery after unilateral stroke. | 30 total, 10 in the mCIMT group, 10 in the ICR and 10 in the CR group. | Participants in the CR group received 45  minutes of consecutive occupational therapy (OT) sessions, 5 days per  week for 4 weeks. Participants exposed to ICR  received 3 h of consecutive OT sessions, 5 days per week for 4 weeks. Participants in the  mCIMT group received 3 h of consecutive OT sessions, 5 days per  week for 4 weeks | - Improvement in motor function of the affected upper extremity did not differ significantly between patients who received mCIMT and those who received ICR.  - mCIMT had better therapeutic effects in improving motor function of patients with acute and subacute stroke than CR therapy. |
| Lynne V. Gauthier; Edward Taub; Christi Perkins; Magdalene Ortmann; Victor W. Mark; Gitendra Uswatte, 2008. | Test the importance of different components of CIMT therapy | 36 total, 16 in the CIMT group and 20 in the comparison group. | Patients randomized to CIMT therapy received intensive in-laboratory training of the more  impaired arm on functional tasks for 3 hours daily for 10 consecutive weekdays, restraint of  the less-impaired arm for a target 90% of waking hours, and a number of behavioral techniques  termed the “transfer package” lasting an additional 0.5 hours in the laboratory. | - Three different analyzes provided converging evidence that the group receiving CI therapy showed profuse changes in gray matter in sensory and motor areas of the brain and hippocampus, accompanied by large improvements in real-world spontaneous arm function.  - This study shows that a rehabilitation intervention can result in structural reorganization in damaged human brains and that the magnitude of this structural change is directly proportional to the amount of clinical improvement. |
| Kate Hayner; Ginny Gibson; Gordon Muir Giles, 2010 | Contribute to the understanding of CIMT, comparing two treatments for chronic UE dysfunction in people in the post-stroke period. | 12 total | 6 hr of occupational therapy for 10 days plus additional home practice | - CIMT is effective in increasing UE movement in post-stroke people with chronic UE dysfunction. Both CIMT and bilateral groups showed statistically significant improvements over the study period.  - Intensive occupational therapy provided in a CIMT or bilateral format appears to be effective in improving motor function in the EU of post-stroke people with chronic EU dysfunction. |
| Ching-yi Wu; Keh-chung Lin; Hsieh-ching Chen; I-hsuen Chen; Wei-hsien Hong, 2007. | To verify whether stroke patients receiving mCIMT, compared with patients receiving traditional rehabilitation (TR), would exhibit better motor control performance and obtain higher scores in the affected UE during unilateral and bimanual tasks. | 30 total, 15 in the mCIMT group and 15 in the TR group | Training was administered intensively  2 hours per day, 5 days per week, for 3 weeks | - mCIMT was associated with greater improvement in range motor control and functional performance than TR.  - mCIMT was associated with greater improvement than TR in daily functioning, but also in motor control. |
| Muhammad Aliyu Abba; Abubakar Shuaibu Muhammad; Umaru Muhammad Badaru; Auwal Abdullahi, 2020. | To determine the comparative effect of CIMT and PNF on upper limb recovery. | 30 total, 15 in the CIMT group and 15 in the PNF group. | Group A received CIMT for 45 minutes, with the unaffected  upper limb restricted by using triangular bandage, by a trained  and experienced physiotherapist. The intervention was conducted 3 times a week for a period of 6 weeks  Group B received PNF intervention administered for 45  minutes to the upper limb 3 times a week for a period of 6 weeks. | - A significant post-intervention improvement in upper limb function in the PNF group. A significant post-intervention improvement in upper limb function in the CIMT group.  - Both PNF and CIMT were effective in the management of post-stroke function of the upper limbs; however, CIMT may be the preferred technique for upper limb function recovery. |
| Nicola Smania; Marialuisa Gandolfi; Stefano Paolucci; Marco Iosa; Patrizia Ianes; Serena Recchia; Chiara Giovanzana; Franco Molteni; Renato Avesani; Pietro Di Paolo; Massimo Zaccala; Michela Agostini; Cristina Tassorelli; Antonio Fiaschi; Daniela Primon; Maria Grazia Ceravolo; Simona Farina, 2012. | To verify whether an mCIMT program can induce greater improvement in the function and use of the affected arm than a conventional rehabilitation program used for stroke patients in Italy. | 36 total | Each group underwent 10 (2 h/d) treatment sessions (5 d/wk for 2 weeks) | - They suggested that an mCIMT training program could improve the function and use of the affected arm more than a conventional rehabilitation program in outpatients with chronic stroke.  - The mCIMT protocol used may be more effective compared to conventional rehabilitation in patients with chronic stroke |
| Jeong-Hui Kim; Moon-Young Chang, 2018. | To investigate the effects of mCIMT on upper extremity function and occupational performance in stroke patients. | 14 total, 7 in the experimental group and 7 in the control group. | The hemiplegic upper extremities in the mCIMT group were trained for 5 days/week, during  the period of 2 weeks by a licensed occupational therapist. To apply the mCIMT, a group underwent 2 hours per day of  adaptive task practice and task training of the paretic limb. In addition, patients wore mitt glove on the unaffected hand for  nearly 6 hours of walking. | - The mCIMT group showed improvements in occupational performance and also in upper limb function than the CRT group.  - Validate that mCIMT is effective in the occupational performance of stroke patients, as well as in the function of the upper limbs. |
| Iris Charlotte Brunner; Jan Sture Skouen; Liv Inger Strand, 2012. | To compare the efficacy of modified restriction-induced movement therapy with combined-dose bimanual task-related training for patients in the subacute post-stroke phase. | 28 total, 13 in the induced movement therapy group and 15 in the bimanual training group. | They were expected to use the  affected arm actively at least 2–3 hours a day. Patients in the modified constraint-induced  movement therapy group were supposed to wear a  mitt on the less affected arm for 4 hours a day and  record the time of actually wearing the mitt in their  logbook | - No superiority of the modified restriction-induced movement therapy approach over bilateral task-related training in improving arm function in the subacute post-stroke phase was found.  - Modified restriction-induced movement therapy is no more beneficial than bilateral training in the subacute post-stroke phase. |
| Dae-Hyouk Bang, 2016. | To investigate the effectiveness of mCIMT combined with auditory feedback for trunk control in moderately impaired UE and ADL function among patients with subacute stroke using the randomized controlled trial protocol. | 20 total, 10 in the mCIMT combined with auditory feedback and 10 in the mCIMT group. | Each participant in both groups received an hour of  individual treatment and engaged in 5 hours of household activities 5 days/wee. During  the 4-week period, the participants’ less affected hands  and wrists were placed in mitts with self-adhesive straps  every day for 5 hours (except Saturdays and Sundays) | - mCIMT combined with auditory feedback leads to a significant improvement in UE function compared with mCIMT alone among moderately impaired subacute stroke patients.  - mCIMT combined with auditory feedback can improve UE function, with potential applications in clinical rehabilitation. This combined training method also appears to have a positive effect on UE motor function and ability to perform ADL |
| Masahiro Abo; Wataru Kakuda; Ryo Momosaki; Hiroaki Harashima; Miki Kojima; Shigeto Watanabe; Toshihiro Sato; Aki Yokoi; Takuma Umemori; Jinichi Sasanuma, 2014. | To compare the clinical efficacy of these two interventions (NEURO versus CIMT) for upper limb hemiparesis after stroke. | 76 total, 38 in the NEURO arm group and 38 in the CIMT group. | NEURO group:22 sessions of 20-min LF-rTMS,  60-min one-to-one training and 60-min self-training for 15 days. CIMT group:  11 sessions of 6-hour constraintinduced movement therapy with supervisors for 15 days | - Significant differences both in the FMA, which assesses the entire arm, and in the FAS, which reflects the ease of use of the arm in activities of daily living. Thus, the results suggest that the improvement in whole arm movement after NEURO is superior to that after CIMT.  - Superiority of NEURO in relation to CIMT. The preferred outcome after NEURO may be due to improved movement of the entire upper limb with resulting functional improvement in activities of daily living. |
| Hee Kim; Eun-Young Yoo; Min-Ye Jung; Jongbae Kim; Ji-Hyuk Park; Dae-Hyuk Kang, 2017. | To compare the effects of combination therapy of mCIMT with mental practice versus those of mCIMT alone on change in corticospinal excitability, quality of movement of the affected upper extremity, higher motor functions, and performance of the affected arm in daily life. | 14 total, 7 in the experimental group and 7 in the control group | Participants to perform their everyday activities while wearing a hand constraint for more than  six hours a day five days per week for two weeks. During these  six hours, participants had to visit the therapist and repeatedly  practice ADL tasks with therapist supervision for one hour per  day. The mental practice coupled with action observation was performed by listening to audio material while watching a video for  10 min. | - Both groups demonstrated improvement in movement quality and ADL functions on the affected side.  - The combined therapy of mental practice and mCIMT proved to be an effective intervention method for stroke patients, showing effectiveness in a short period of time |
| Dae-Hyouk Bang; Won-Seob Shinb; Ho-Suk Choib, 2018. | To explore the effectiveness of mCIMT combined with RT on upper limb function and ADLs in early stroke patients using a randomized controlled trial (RCT) protocol. | 24 total, 12 in the CIMT + TR group and 12 in the mCIMT group. | In both groups, the training was provided at the  rehabilitation clinic during a 1-hour therapy session,  5 days per week, for 4 weeks (a total of 20 sessions) | - mCIMT combined with RT may be more effective than mCIMT alone in improving upper limb function and ADLs in early stroke patients.  - mCIMT combined with TR improves upper limb function and suggests the applicability of mCIMT combined with TR for clinical rehabilitation. |
| K.-C. Lin; C.-Y. Wu; T.-H. Wei; Chang Gung; C.-Y. Lee; J.-S. Liu, 2007. | To verify if modified restriction-induced movement therapy would have beneficial effects on both functional capacity and control of reach and grip. | 32 total, 17 in the induced movement therapy group and 15 in the traditional rehabilitation group. | Restriction of movement of the unaffected hand by placement in a mitt for 6 hours  per day and (2) intensive training of the affected  arm for 2 hours per weekday. The traditional rehabilitation was designed  to control for the duration and intensity of  patient–therapist interaction and therapeutic  activities (5 days/week for 2 hours/day for three  consecutive weeks) | - Modified restriction-induced movement therapy induced more efficient pre-planning of reaching and grasping and a shift towards feedforward control over traditional rehabilitation.  - Greater improvements in functional performance of daily activities and motor control during reach-to-grasp movements after modified constraint-induced movement therapy versus traditional rehabilitation. |
| Karen Atler; Matthew Malcolm; Catherine Greife, 2015. | Address participation by following stroke survivors who had previously received CIMT, examining the question: what is the relationship between upper limb motor function, activity and participation? | 12 total | They  were asked to complete the diary Profile for three days within the  next week. During the participant-selected weekend day and two  week days, the participant stopped two to three times throughout  his or her day to record activities and rated his or her experiences  of pleasure, productivity and restoration for each activity. | - For three of the main motor function and activity measures (ie WMFT time, WMFT functional ability and MAL how well), participants demonstrated significant improvements from pre to post-CIMT.  - Increased motor or activity performance does not suggest greater participation in any specific activity category, although there is a correlation with time spent with other people, which supports the ICF model. |
| Jin A Yoon; Bon Il Koo; Myung Jun Shin; Yong Beom Shin; Hyun-Yoon Ko; Yong-Il Shin, 2014. | To investigate the superiority and synergistic effect of CIMT combined with mirror therapy for the improvement of gross and fine motor functions of the hemiplegic upper extremity. | 26 total, 8 in the CIMT combined with mirror therapy group, 9 in the CIMT-only group and 9 in the control group. | The patients received  intensive training for five days a week except for the  weekend, for a total of six hours (2 hours in the therapy  room and 4 hours in the inpatient room) a day except for  sleeping hours | - Results indicate that mirror therapy combined with CIMT would be effective in improving fine motor function indicators in the clinical setting.  - CIMT combined with or without mirror therapy groups showed more improvement than the control group on most functional assessments in the hemiplegic upper extremity. The CIMT group combined with mirror therapy achieved more significant improvement than the CIMT group only on the box and block test, 9-hole Pegboard test, and grip strength, which represent fine motor functions of the upper extremity. |

Legend: CIMT: constraint -Induced Movement Therapy; EP: Technique of occlusion of one of the eyes using an eye protector; MAL: Motor Activity Log; dCIMT: Distributed Contra-Induced Movement Therapy; CI: Control Intervention; FMA: Fugl-Meyer Assessment; UE: Upper E ADL: Activity of Daily Living; mCIMT: Modified constraint -Induced Movement Therapy; MP: Mental Practice; BAT: Bilateral Arm Training; UL: upper limb; MS: Senior Member; CVA: Cerebral Vascular Accident; QOL: Quality of Life; ICR: Intensive Conventional Rehabilitation; RC: Conventional Rehabilitation; WMFT: Wolf Motor Function Test; PNF: Proprioceptive Neuromuscular Facilitation; CRT: Conventional Rehabilitation Therapy; FAS: Functional Ability Score; TR: Trunk Restriction; MMSE: MiniMental Sate Examination; MFT: Manual Function Test; K-MBI: Korean version of the Modified Barthel Index; ARAT: Action Research Arm Test; FIM: Functional Independence Measure; SIS: Stroke Impact Scale; BI: Barthel Index; mBI: modified Barthel index; PPR Profile: Profile of Daily Experiences of Pleasure, Productivity and Restoration.
